# Aquatic behaviour of polar bears (*Ursus maritimus*) in an increasingly ice-free Arctic

**DOI:** 10.1038/s41598-018-27947-4

**Published:** 2018-06-26

**Authors:** Karen Lone, Kit M. Kovacs, Christian Lydersen, Mike Fedak, Magnus Andersen, Philip Lovell, Jon Aars

**Affiliations:** 1 Norwegian Polar Institute, Norwegian Polar Institute, Fram Centre, 9296 Tromsø, Norway; 2 Sea Mammal Research Unit, Gatty Marine Laboratory, University of St Andrews, St Andrews, Fife KY16 8LB UK

## Abstract

Polar bears are ice-associated marine mammals that are known to swim and dive, yet their aquatic behaviour is poorly documented. Reductions in Arctic sea ice are clearly a major threat to this species, but understanding polar bears’ potential behavioural plasticity with respect to the ongoing changes requires knowledge of their swimming and diving skills. This study quantified time spent in water by adult female polar bears (n = 57) via deployment of various instruments bearing saltwater switches, and in some case pressure sensors (79 deployments, 64.8 bear-years of data). There were marked seasonal patterns in aquatic behaviour, with more time spent in the water during summer, when 75% of the polar bears swam daily (May-July). Females with cubs-of-the-year spent less time in the water than other females from den emergence (April) until mid-summer, consistent with small cubs being vulnerable to hypothermia and drowning. Some bears undertook notable long-distance-swims. Dive depths up to 13.9 m were recorded, with dives ≥5 m being common. The considerable swimming and diving capacities of polar bears might provide them with tools to exploit aquatic environments previously not utilized. This is likely to be increasingly important to the species’ survival in an Arctic with little or no persistent sea ice.

## Introduction

Polar bears are strong swimmers^1,2^ that are classified as marine mammals because of their strong dependence on marine food resources. This species hunts ice-associated seals (and whales) in areas with land-fast ice or drifting pack-ice and is therefore associated with environments that are naturally a mixture of open water and sea ice. Polar bears also hunt on land, or from shore, when there is little or no ice. During such times, swimming can be the easiest (or only) way to get to a desired destination, either because water must be crossed to achieve the shortest route, or because swimming might allow them to avoid difficult terrain (e.g. coastal cliffs, glacier fronts), or because the destination is an island surrounded by open water. They also swim and dive to: (1) approach seals resting on ice floes^1^; (2) hunt birds^3^, fish^4^, beluga or narwhal^5^ and; (3) access other marine resources such as seaweed^1,6^ or sunken cadavers. Polar bears also go into the sea to clean themselves or to cool down. In short, there are many reasons why polar bears spend time in the water and not surprisingly they are very comfortable in this environment and are clearly well adapted to it^1^.

Sea ice is an important platform for polar bear breeding and hunting^7^, and it serves as a transit corridor between denning, hunting and breeding sites when these areas are separated spatially^8^. Polar bears sometimes make transits across significant open water areas and consequently swim long distances^2,9^. While polar bears are very capable swimmers, such long-distance swims can have negative consequences, including increased mortality in the extreme^10,11^. Females accompanied by small cubs are particularly dependent on sea ice being available during offshore transits^12,13^. This is because the young cubs of the year (COYs) are initially not well adapted to spending time in the water and will become hypothermic relatively easily because they have little insulating fat^14,15^.

Polar bears in the Barents Sea subpopulation use two main habitat strategies. ‘Local’ polar bears stay on land or in coastal areas near Svalbard year round, and ‘offshore’ polar bears use the offshore pack ice much of the year, returning to the islands within the Svalbard or Franz Josef Land Archipelagos for denning^16–18^. Current declining trends in sea ice in Svalbard are predicted to increase the need for polar bears to become more aquatic, since the distance between denning areas and the polar cap’s sea ice is increasing and the duration of the ice-free season around Svalbard is also increasing.

Polar bears hunt both in drift ice, and on annually formed ice, where bears either actively crush lairs to access ringed seals, or rush them on the ice surface, or stalk them by sitting at breathing holes and pouncing on the seals when they surface to breathe. However, some bears also hunt by ‘aquatic stalking’ of seals that are hauled out on ice in areas with a lot of open water^1,19^. Aquatic stalking seems to be a more specialized technique than the widely used still-hunting techniques, and only some polar bears are thought to master it^1,20^. However, in areas where stable sea ice platforms are not available, and seals haul out on small pieces of sea ice or glacier bits, this is perhaps the only strategy that works. Being able to dive is an important skill in this context, as it allows the polar bear to do the final approach completely out of sight of the hauled out seal. The diving capabilities of polar bears are not well known, although some fragmentary information can be found in the literature. Stirling^1^ recorded submergence times for four dives: 37, 40, 55, and 72 s. He also reported a polar bear approaching a bearded seal over a distance of 300 m by conducting a series of dives^1^. The longest submersion thus far recorded for a polar bear is 3 min and 10 s^19^; this dive also occurred as part of an aquatic stalk of a bearded seal.

Although polar bears are known to be semi-aquatic, there is little hard data on this aspect of their ecology. Direct observations by Stirling^1^ indicated that polar bears at Devon Island in Baffin Bay, Canada, swam 4.1% of the time during midsummer. Other studies have reported polar bears’ abilities to undertake long-distance swims. These swimming records have been based on data from tracking and sea ice data, in combination with an internal logger measuring body temperature^2,10^, or with evidence of swimming being based on gaps in location records that are assumed to be due to failure to acquire Argos locations (or send GPS-positions) because the tracking device’s antenna is submerged in water^9^. The purpose of the present study was to quantify the aquatic behaviour of adult female polar bears captured in Svalbard using various biologging instruments equipped with saltwater switches and pressure sensors (Table 1; additional details in Supplementary Information Appendix 1). Individual, seasonal, and geographical (local vs offshore ecotype) differences in aquatic behaviour are reported, with a particular focus on time spent in the water, and whether swimming was correlated with sea ice concentrations or reproductive status (i.e. whether females were accompanied by cubs).

**Table 1:** Sensor information for the three telemetric devices used to collect data on swimming (and diving) behaviour of polar bears.

Tag manufacturer and model	Device functioning with respect to swimming and diving data	Deployment period	Number of tags that provided data	Length of records (days), mean ± sd, [range]
Wildlife Computers’ TDR-Mk9	Time-depth-recorder; an archival tag without any onboard algorithm. Stored wet/dry, depth, light, temperature sensor readings every 10 seconds. Wet/dry readings below 100 (well above reading of sea water) were later summed to give % time in water hourly	2010–2017	29	372 ± 266, [69–1239]
Telonics’ TGW-4678–3 and TGW-4678-4	Collar with integrated sensors and software; data transmission via Iridium. Swims start with 5 seconds wet and end with 1 dry second. Transmitted hourly summaries of % time in water.	2015–2017	31	320 ± 211, [40–736]
SMRU’s Argos SRDL	Collar with integrated sensors and software; data transmission by Argos. Swims start with 12 seconds wet and end with 1 dry second. Stored 4 h summaries of % time in water, max depth, average duration and max duration, as well as time, duration and max depth of individual swims. Transmitted a random subset of the data. Recovered instruments contain the full data set(s).	2005–2008	Only Argos-transmitted: 13 Recovered: 6	Only Argos-transmitted: 123 ± 114, [0,359] Recovered: 678 ± 321, [364–1278]

## Results

### Tag function and data retrieval

**Figure 1:**
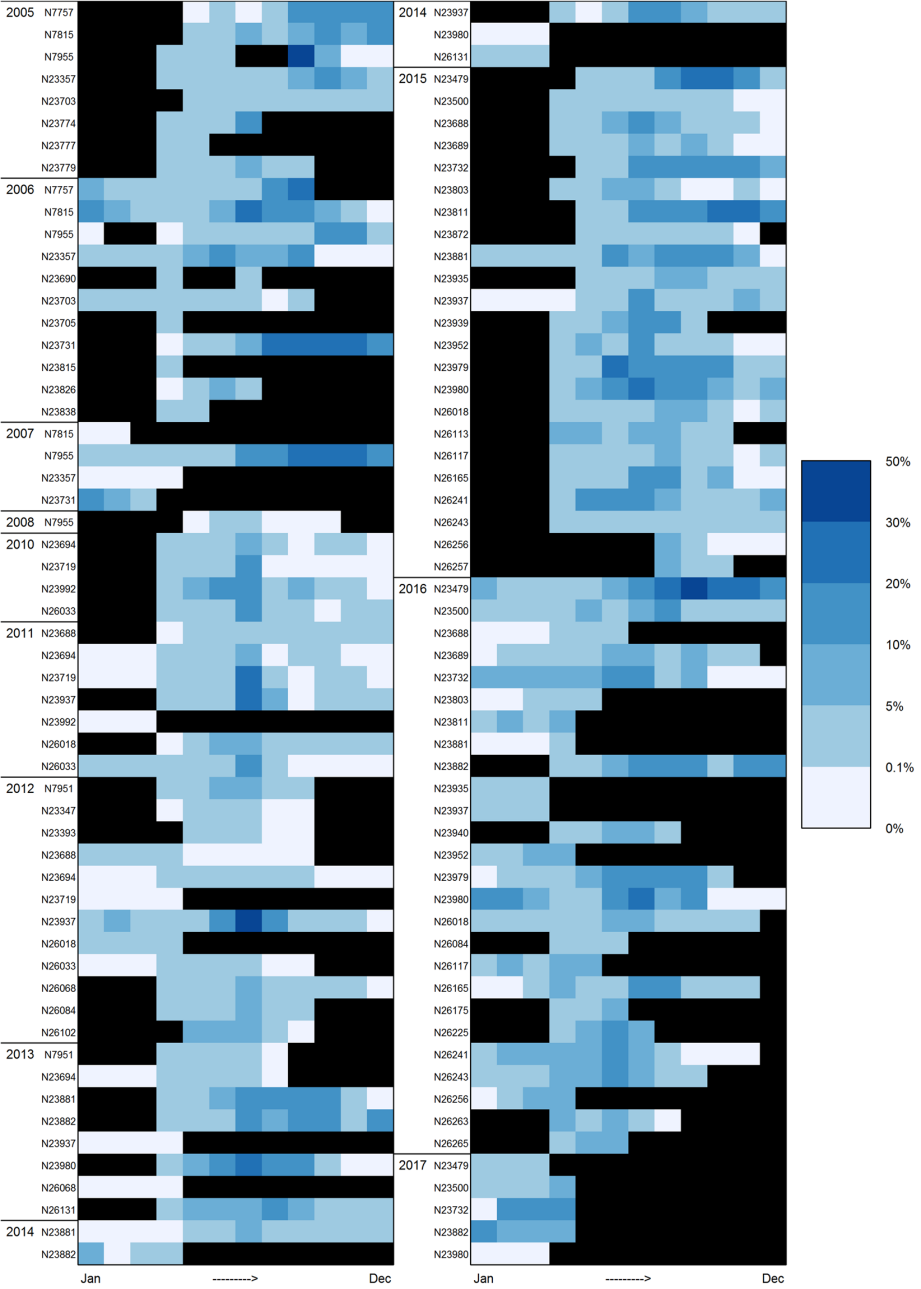
Seasonal and individual variation in percent time in water for female polar bears in the Barents Sea subpopulation. Monthly means (based on minimum 5 days of data) for each individual ordered by year (black blocks indicate no data).

In total, 11.6 years of data from SMRU collars, 29.6 years of data from Wildlife Computers TDRs, and 27.1 years of data from Telonics collars were collected. Excluding the overlap between the latter two, this totalled 64.8 years of data on polar bear use of aquatic habitats. In total 818 monthly average values of percent time in water (based on 5 days or more) were analysed (Fig. 1), with 54 of the 57 bears contributing data (the last 3 did not have any months with 5 days or more of data). Approximately half of the swimming data could be associated with measured or interpolated locations (147,121 polar bear locations with known time in water for a 2 hr period, centred on the location; see Appendix 2 Fig. S2 for geographical distribution).

### General description of aquatic behaviour

**Figure 2:**
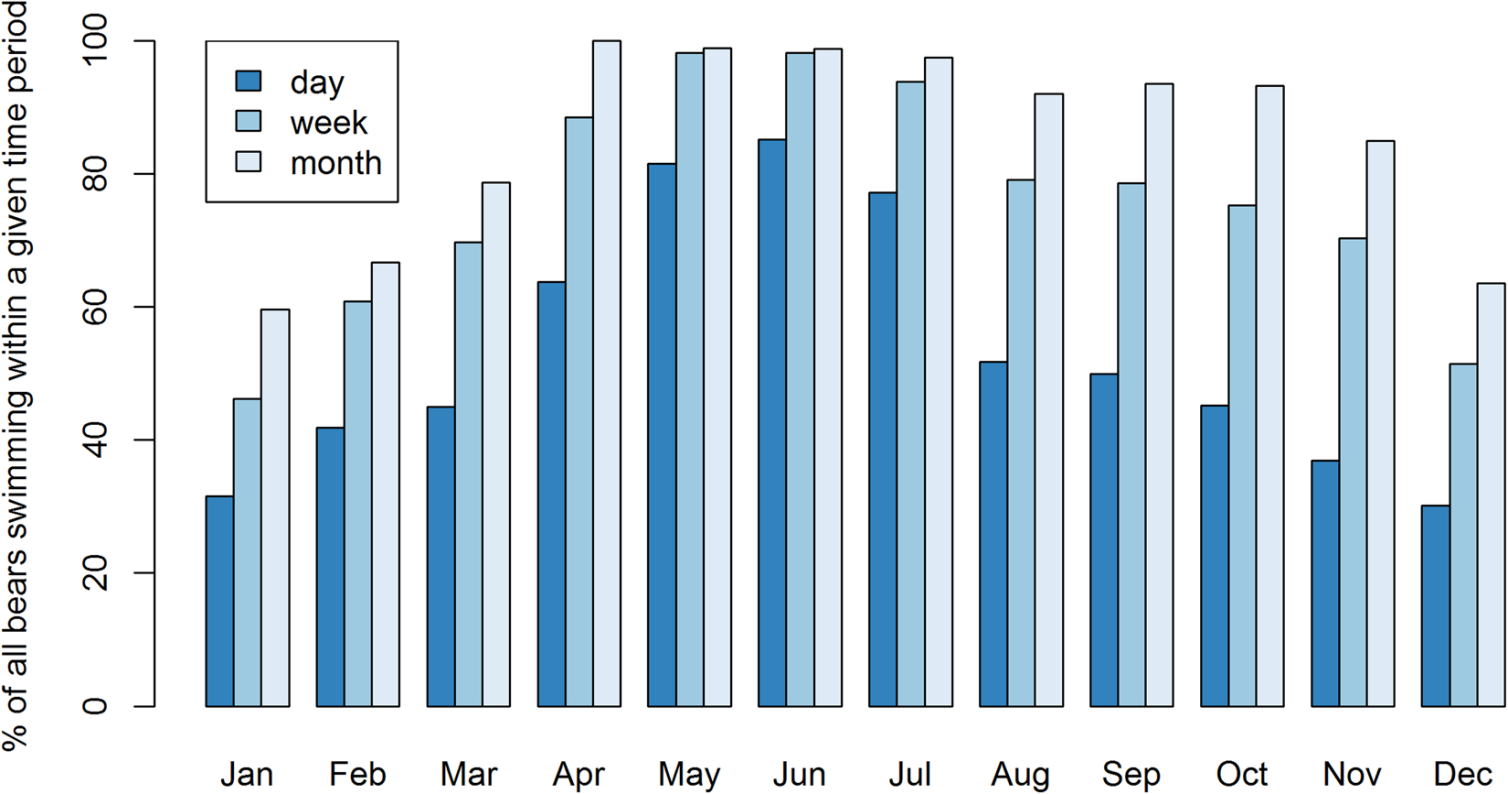
Proportion of instrumented polar bears (all sensor types) exposed to water (i.e. swimming) in the course of 24 hrs, 1 week or 1 month according to month.

All of the polar bears spent some time in the water (Fig. 1). However, there was large individual variation in the amount of time spent swimming. There were also marked seasonal patterns within and across individual records. Many of the records included a winter period with no swimming corresponding to the months that a reproductive denning period would typically last (November/December through March), and all polar bears swam more in summer and early fall than in the winter and spring (Fig. 1). In the course of one day, the percentage of instrumented polar bears that swam was above 30% during all months (Fig. 2), and during May, June, and July, over 75% of the daily records included swimming. Considering monthly records, over 90% of the polar bears swam in April to October. The percentage of polar bears that swam in the course of a month declined to about 60% for a period from December to February.

**Figure 3:**
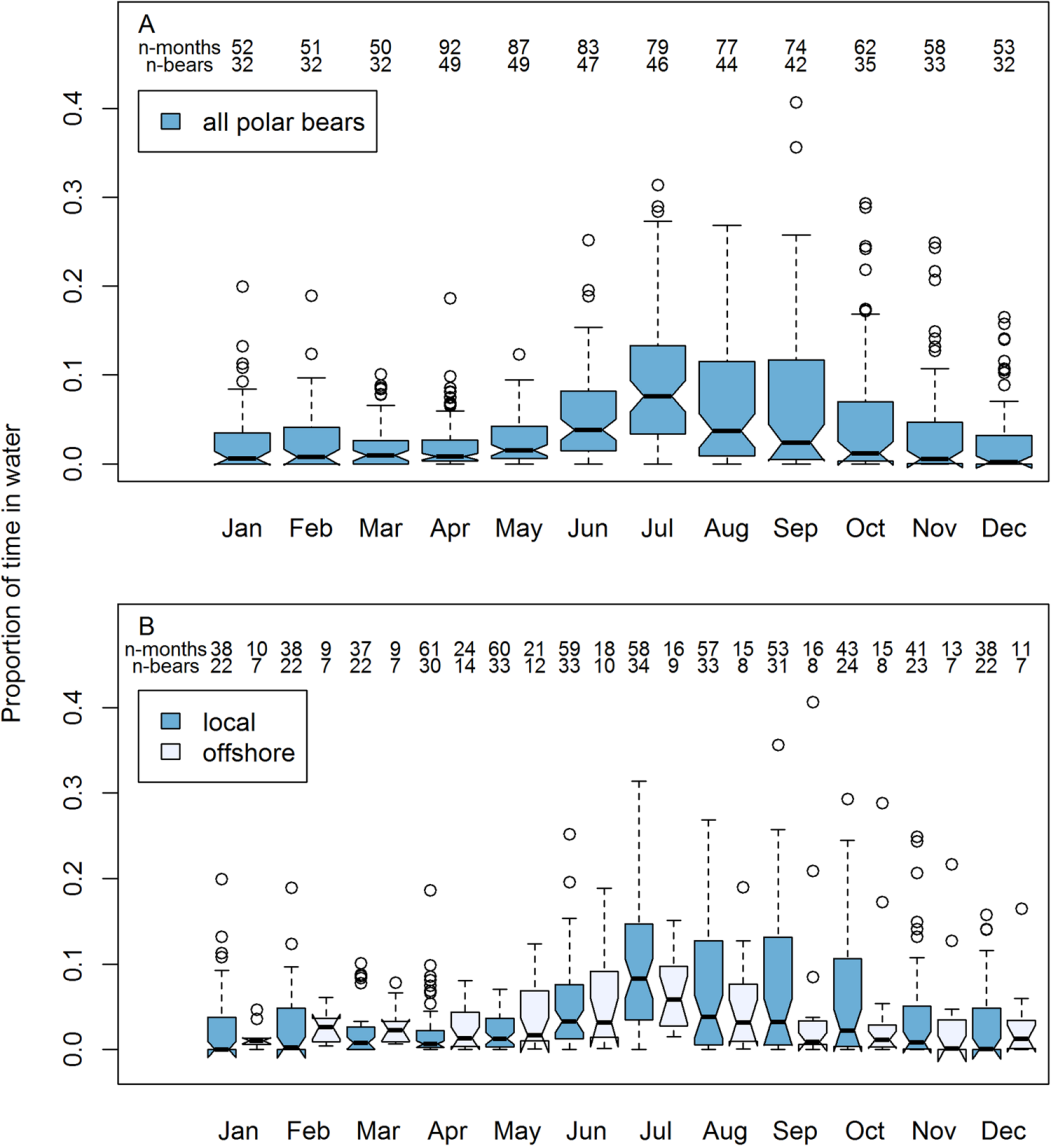
Mean proportion of time spent in the water by month (**A**) and space use strategy (**B**: ‘offshore’ encompasses data from polar bears that had made an offshore excursion at some point during tracking, while ‘local’ polar bears had not) for female polar bears in the Barents Sea subpopulation. Plotted values are monthly aggregates for individual polar bears (N given as ‘n-bears’), with polar bears tagged in multiple years contributing multiple monthly values (N given as ‘n-months’).

The marked seasonal pattern in aquatic behaviour at the individual level was also reflected at the population level, with polar bears generally spending more time in water in summer (June–August) than at other times of the year (Fig. 3A). The lowest monthly mean time in water occurred in March (2.0%, range 0.0–10.0%) and the highest in July (9.4%, range 0.0–31.0%). No significant differences were found between local and offshore polar bears with respect to monthly mean time spent in the water (Fig. 3B; Supplementary Information Appendix 2 Table S1; based on 760 months of data from 49 individuals). Offshore polar bears may swim more than local polar bears in the transition period between winter and spring and local polar bears may swim more than offshore polar bears during summer, but the statistical support for these trends was weak (May, p = 0.04, and July, p = 0.1; Supplementary Information Appendix 2 Table S1).

**Figure 4:**
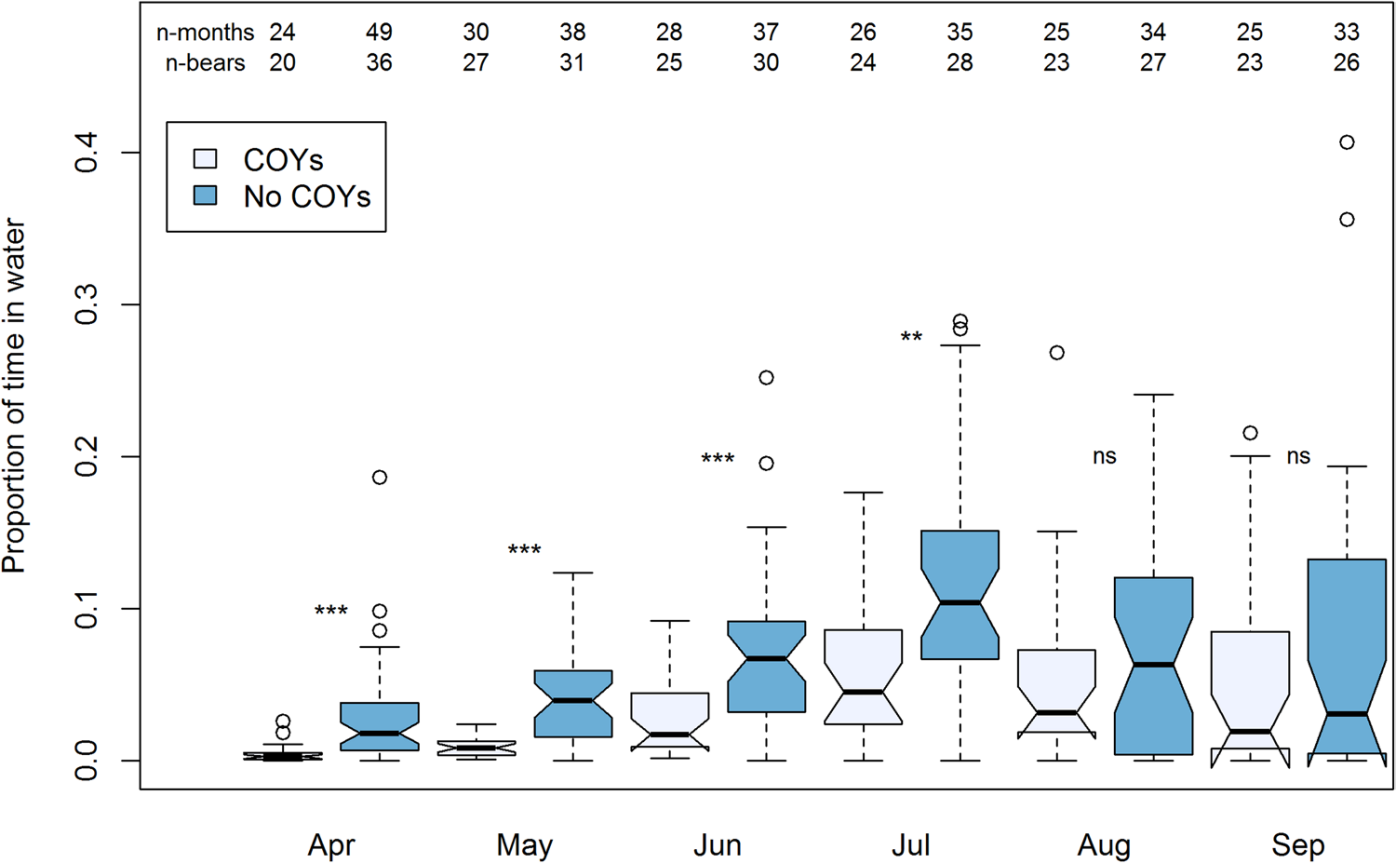
Mean proportion of time spent in water by month and reproductive status (with vs without cubs of the year (COYs)) for female polar bears in the Barents Sea subpopulation. Plotted values are monthly aggregates (N given by ‘n-months’) for individual polar bears (N given as ‘n-bears’), with polar bears tagged in multiple years contributing multiple monthly values.

Reproductive status of the female polar bears had profound effects on the amount of time they spent in the water during the summer months. Females with yearlings and females without cubs showed no overall differences (Supplementary Information Appendix 2 Table S1), so these reproductive classes were pooled in the analyses. Females with COYs spent less time in the water than the other reproductive class from April (den emergence) until July (Fig. 4; based on 384 months of data from 54 adult female polar bears). The difference between the two groups decreased with time, and by August they were no longer statistically different (Fig. 4, Supplementary Information Appendix 2 Table S1).

**Figure 5:**
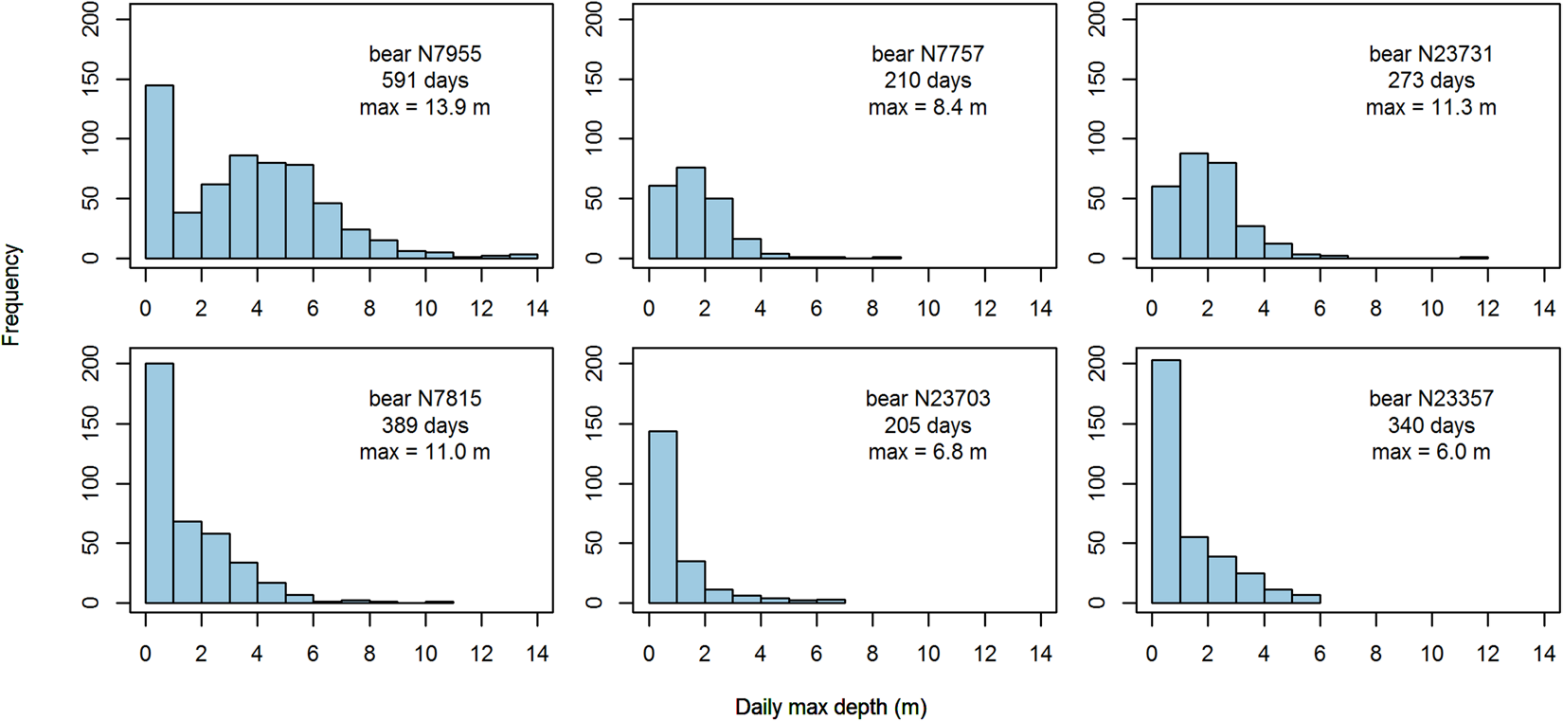
Histograms and summary statistics of daily maximum dive depth for all days with registered swims from six polar bears whose complete diving record were downloaded following SMRU collar recovery.

Maximum daily dive depths for six polar bears for which complete diving records were available are presented in Fig. 5. The polar bears stayed at the surface most of the time when they were swimming; 43 ± 20% days with registered swimming had max dive depths between 0 and 1 m. However, all six polar bears dove to 3–4 m on many occasions. A 14 year old female (N7955) that did not have cubs when the collar was deployed, nor when she was recaptured three years later, dove deeper and more often than the other polar bears. This polar bear had few days with only surface swimming. She dove to intermediate depths frequently, and on multiple occasions she dove deeply (≥8 m on 36 different days, ≥10 m on 11 different days; max depth 13.9 m). Only a few of her diving events have associated location data, but these included deep dives (<8 m) made at locations both in drift ice offshore and at a coastal location. Considering all the polar bears with more than 100 recorded swimming events (transmitted by Argos or downloaded after recapture), 13 of 14 polar bears dove to ≥6 m.

**Figure 6:**
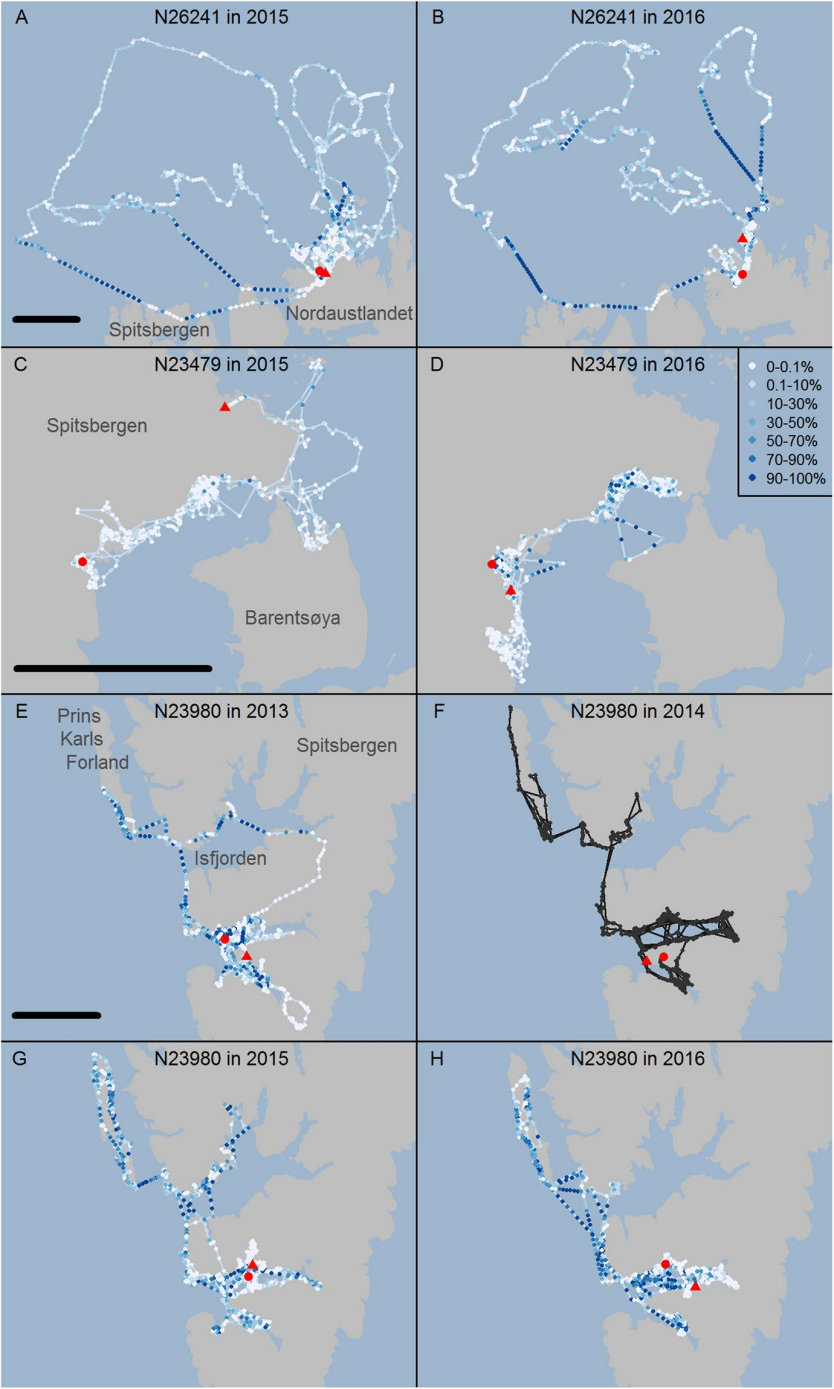
Example tracks from three female polar bears: N26241 made several excursions from North Nordaustlandet into the MIZ in 2015 (**A**) and 2016 (**B**); N23497 used the same area on the eastern coast of Spitsbergen in April-July while accompanied with COYs in 2015 (**C**) and with yearlings in 2016 **(D**); and N23980 used the same area on the western coast of Spitsbergen without cubs in 2013 (**E**) and 2016 (**H**), with COYs in 2014 (**F**) and yearlings in 2015 (**G**). Red circles and triangles mark beginning and end of tracks, respectively. Locations are either recorded or linearly interpolated within gaps up to 48 h. Color of the locations represents proportion time in water over a two hour period centered on the time of location. Black bar indicate scale (50 km) for plots for each bear. For track F, time in water was not available (the TDR fell off the collar), a GPS track without swimming data is shown for comparison with the other years for this polar bear. The maps were created in R 3.2.2^35^ and the underlying map data are available from http://geodata.npolar.no, ©Norwegian Polar Institute.

Data from three polar bears (one offshore and two local) illustrate the individual variation in swimming performance and strategies (Fig. 6). The first of these example polar bears, N26241, used the offshore Marginal Ice Zone (MIZ) and undertook several long transit swims between the MIZ and land (Fig. 6A,B). This bear used areas north of Spitsbergen and Nordaustlandet in 2015 and 2016. She was 8 years old when she was collared in 2015, and did not have cubs in either year. This bear exemplifies the capacity of some polar bears in the Barents Sea subpopulation to make repeated long-distance swims. In 2015, she transited twice from the MIZ to North Spitsbergen (18 May: 70 km, swimming 26 h of 28 h, 24 h continuously; 6 June: 92 km, swimming 30 h of 38 h, 18 h continuously). In 2016, her three longest swims were from the MIZ to North Spitsbergen (14 June: 57 km, swimming 36 h of 42 h, 18 h continuously), from Nordaustlandet to the MIZ (29 June: 49 km, swimming 19 h of 28 h, 10 h continuously), and from the MIZ to Nordaustlandet on 9 July, which took place in two spurts separated by a 9 h break (34 km, swimming 13.5 h of 17 h, and 65 km, swimming 35 h of 42 h, 34 h continuously).

The second example polar bear, N23479, illustrates how female polar bears reduce their swimming during the months April-July when accompanied by COYs (Fig. 6C,D). In 2015, this polar bear was 17 years old and had two COYs, and swam much less than she did the following year, when her cubs were yearlings. However, towards the end of the first summer with the COYs, she was already swimming much more, with the longest swim recorded being 6 hours, and time in water averaging between 14 and 24% for the months August-November. The family group used the same geographical area in both years, and both cubs survived the two years. The third example polar bear, N23980, illustrates a polar bear that used a special strategy (Fig. 6E,F,G,H). This bear swam regularly between islands and across fjords, even when accompanied by two COYs. She exhibited a set temporal pattern to her space use along the west coast of Spitsbergen, where she moved north from Van Mijenfjorden to Prins Karls Forland and affiliated small islands (PKF), the latter area being the core breeding area for Svalbard’s harbour seal population. Her time of arrival at PKF specifically coincided with the end of the harbour seal nursing period, when newly weaned pups were available (pers. obs. KM Kovacs, C Lydersen) in June-July. In recent years, this bear, which was 7 years old in 2013, undertook this trip annually, irrespective of whether she was alone (2013 and 2016), or with cubs (COYs in 2014, yearlings in 2015). The area was ice-free in summer and thus reaching this small island group required swimming across fjords and stretches of open water. In the years she did not have COYs, she spent between 4 and 7 h swimming to cross Isfjorden (minimum swimming distance 12 km), and between 5 and 11 h swimming crossing over to PKF (minimum swimming distance 17 km). In 2014, when she had COYs, there is no direct measurement of time in water, as the TDR was not recovered, but she moved along the same route and crossed Isfjorden on 22 June, crossed to PKF on 12 July, and made the return trip on 10 August. The gaps in the GPS track when the polar bear crossed to and from PKF were 8 h and 10 h, respectively, suggesting that the durations of these swims were similar to the other years. Of the eight females with COY(s) that were later captured and therefore known to have survived their first year, for which there is aquatic data, the longest swim recorded is of similar length: She made an 8.3 hr long swim in the end of June, and another 8.4 hr swim in August.

### Modelling aquatic behaviour in relation to environmental parameters

Temporal trends were present in some of the swimming measures. Julian day had a significant effect on the probability of swimming when considering records with any time in water in 2 h periods (GAMM: p < 0.001) or with a 1 min swimming threshold (GAMM: p < 0.001). However, it did not have a significant effect on time spent in the water (GAMM: p = 0.405), the probability of swimming for 10 min or more in a 2 h period (GAMM: p = 0.98) or the probability of swimming 60 min or more in a 2 h period (GAMM: p = 0.31). The fitted effect of Julian day was a linear increase with time in all models (edf = 1).

**Figure 7:**
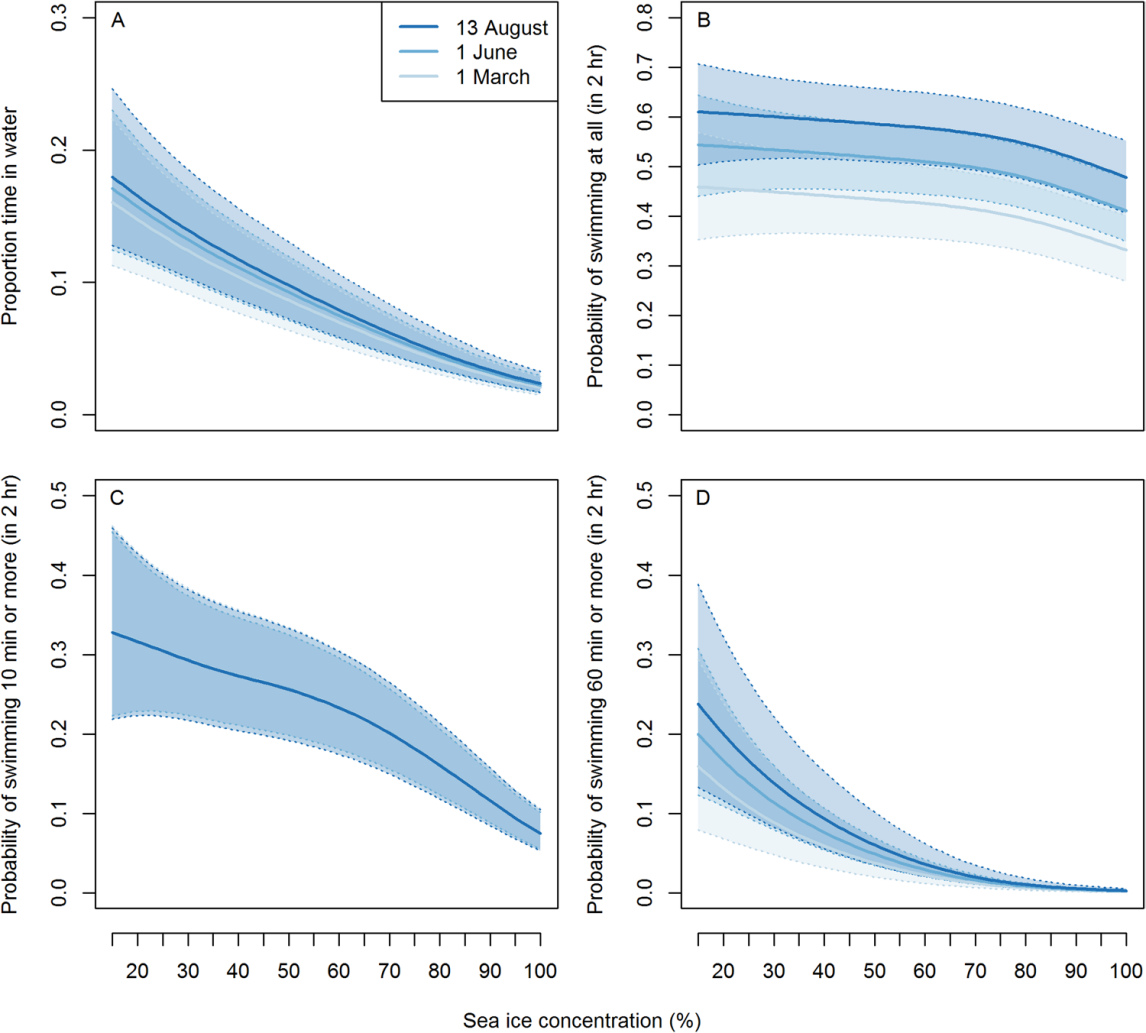
Modelled predictions for time spent in the water (**A**) and the probability of being in water (at all (**B**), or more than 10 min (**C**) or 60 mins (**D**) in a 2 hr period) depending on sea ice concentration. Shaded 95% confidence intervals are presented around the prediction lines.

Proportion of time in water, probability of swimming at all in a 2 hr period, or swimming ≥1 min, ≥10 min or ≥60 min were all dependent on sea ice concentration (GAMM: all p < 0.001). In the model in which both season and sea ice concentration were important, the two variables had a similar effect size on the probability of swimming at all (Fig. 7). The same is true of the model in which swimming ≥1 min was the response variable, which is not shown because it was very similar, just slightly lower, than the probability of swimming at all. Proportion of time spent in the water decreased with total sea ice concentration, from 0.24–0.28 in low sea ice concentrations (here, 15%) to 0.04–0.05 in pixels with close to 100% sea ice cover (Fig. 7, similar to the probabilities of swimming ≥10 min or ≥60 min), with time of year explaining relatively less of the variability in this measure across the period for which we have data (March-August). The probability of swimming at all (in a 2 h period) decreased only slightly with increasing sea ice concentrations, with the rate of decrease becoming somewhat stronger at high sea ice concentrations (Fig. 7).

## Discussion

The results of this study show the importance and pervasiveness of aquatic behaviour for the Barents Sea subpopulation of polar bears. The average percentage of time females swam each month varied considerably from 2.0% in March to 9.4% in July, with large variation between individuals (in July from no time in water and up to 31%). The high variance in the amount of swimming and diving between individuals (in addition to the variance explained by reproductive state) likely reflects individual differences in ecological strategies, and unpredictable environmental variability. Sea ice dynamics in the region are strongly influenced by wind, currents and temperature and there can be large inter-annual variations and day-to-day changes both in drift ice^21,22^ and fast ice^23^ conditions. The time spent swimming, the capacity to dive to >10 m depth, and the long distance swims documented herein all demonstrate that polar bears are well adapted to using Arctic marine environments.

Seasonal variation in sea ice cover is a primary driver that influences swimming behaviour of the polar bears in the Barents Region. The drifting sea ice in this area typically reaches a maximum extent in April and a minimum extent in September^22^. Swimming occurred most commonly during summer and autumn (peaking in July), months with low sea ice cover, and less commonly during the winter and spring when ice cover was greater. The percentage of time that female polar bears swam in July (9.4%) in Svalbard in the current study was substantially higher than what was reported by Stirling^1^ for polar bears on Devon Island in the central Canadian Arctic at the same time of year (4.1%). However, the latter study area was still mainly covered with sea ice in the 1970s and much of the swimming by the bears was done in open pools and leads inside this habitat. During July in Svalbard, most coastal areas have been ice free, except for some pieces of glacier ice. Sea ice conditions in some of Svalbard’s fjords have declined gradually over time, but there was a marked shift to a reduced temporal and spatial extent that occurred in 2006, which has persisted through to the present^23^. The current study mainly documents polar bear swimming behaviour after this shift.

Ringed seals have not altered their habitat selection in response to the sea ice collapse, but polar bear space use during summer has shifted away from glacier fronts^20^, supporting the idea that the hunting strategy required to capture seals using glacier ice is only used by some (likely few) bears. The most extreme cases of swimming behaviour seen in this study, for instance, the polar bear that swam 31% of the time in July (equivalent to swimming 7.5 h every day), may be related to aquatic stalking. The reduced swimming activity by female polar bears in winter is a natural reflection of the fact that approximately 40% of the adult female population goes into dens in late autumn (November-December) through until the spring (March-April)^24^. It may also reflect higher energetic costs of swimming due to low temperatures, or poorer access to prey providing less motivation to swim.

Although polar bears are good swimmers, it is an energetically costly mode of transport. The net metabolic cost of swimming has been estimated to be 5 times that of walking the same distance at the same speed^25^. Maintaining a normal body temperature makes up a large part of this energetic cost, and how costly it actually is depends on body size and body condition as these factors affect heat loss strongly (through the excellent insulative properties of fat and changes in the volume-to-surface ratio). The risk of swimming, especially a long distance, is also related to its energetic cost; the risk of dying from hypothermia or drowning depends on depletion of energy reserves and exhaustion, as well as external factors such as weather and sea state^10,11^.

Even short swims might represent large energetic costs and potentially high risks to COYs, compared to moving over sea ice. COYs are prone to hypothermia due to their small size and little insulating fat^15^, and, considering their young age and lack of muscular development and endurance, they are probably less capable of swimming in rough conditions. The reduced rates of swimming by females with COYs in spring and early summer documented in the present study is likely a risk-reduction mechanism. Lactating females with COYs may also alter their habitat selection to avoid swimming at this time of year in their own interests, as they have gone through a denning period in the winter and are thin upon emergence. Accordingly, females with COYs select areas in bays close to glacier fronts in spring where stable sea ice and easy access to prey (ringed seal pupping habitat) are available, and the threat of cub predation from adult males might be lower^13^. Note, however, that what constitutes a successful strategy of risk management associated with swimming is not easily predicted. In this study, one female bear with two COYs made at least four long swims to migrate to a preferred hunting area on PKF, and both COYs survived all of the swims. The estimated physiological limit for exposure to cold water immediately after den emergence is only 10 minutes^15^, but this likely increases over the summer as COYs grow. However, how high tolerance COYs have for swimming will depend on the condition and development of the cub, and factors such as water temperature, sea state and weather, and behaviours such as piggy-back riding on its mothers’ back which reduces the cub’s exposure to cold water^14^.

When polar bears in the Barents Sea subpopulation are in the MIZ, they show preference for intermediate sea ice concentrations 40–80%^26^, despite the likelihood that they must swim more in such areas compared to areas with higher sea ice concentrations. This is almost certainly due to increased hunting opportunities, which likely offset the cost of swimming, as ringed seals also show preference for intermediate ice concentrations^27,28^. Although it is notable that females with cubs select more solid ice than females without cubs in MIZ areas, similar to coastal areas^12^.

All data presented in the current study relate to adult females, since adult males do not retain neck collars. Relatively few data are available for other sex and age classes of polar bears regarding aquatic behaviour. Stirling (1974) reported that cubs (yearlings and two-year-olds) spent more time in water than adults of both sexes (4.5% vs <1%) during midsummer (July-August) and suggested that they used this time for playing and learning. However, it is not clear what sample size Stirling based his inferences on, and the last age category in his study, ‘adults and subadults of unknown sex’ swam 8.2% of the time, more than any of the other categories, leaving some uncertainty as to what age and sex group actually swam most. There is indirect knowledge about cubs while they are with their mothers, although cubs may swim more or less than their mothers in situations where they have a choice.

Coastal space use was overrepresented in this study: two-thirds of the polar bears were considered to belong to the local ecotype, while this group as a whole constitutes maybe only a tenth of the subpopulation^17,29^. While the analysis indicated broadly similar patterns in the amount and timing of swimming performed by local polar bears and offshore polar bears, differences may have been masked by the large individual variation in aquatic behaviour and relatively small sample for polar bears using the offshore strategy.

This is the first report of the diving capabilities of polar bears based on data from biologging instruments. Most polar bears seldom dive beyond 3–4 m. However, they are clearly physically and behaviourally capable of diving to greater depths. One individual in the study stood out in this regard, by diving more regularly to greater depths. This individual might be a specialist in aquatic stalking, as she also dove when she was offshore in the drifting sea ice. Alongside swimming below ice floes during aquatic stalking, accessing coastal underwater resources such as cadavers or seaweed are likely reasons for the dives made by polar bears in this study. It is well documented that macroalgae is part of the polar bear diet^1,6,30^. Lønø^6^ reported seeing a female polar bear and her yearling cub diving to a depth of 3–4 m in February to retrieve seaweed, which they consumed. The maximum dive depth reported in this study was 13.9 m. Within this depth range, breath-hold abilities are not likely to limit the depth of dives. Dive durations were not extractable in the current study because dive starts and ends were not identifiable in the swimming records, but dives of around one minute have been reported in the literature as being quite normal^1^, with the longest reported dive being 3 min 10 s^19^.

The Barents Sea subpopulation of polar bears has experienced the largest reduction in sea ice of all the subpopulations of polar bears throughout the circumpolar Arctic in recent decades^31^. The extended ice-free season has already had clear effects on strategic choices by polar bears such as their choice of denning areas, with traditional core denning habitats on some islands now rarely being accessible for use due to late arrival of winter sea ice or poor sea ice conditions^32,33^. While polar bears in Svalbard do swim between the islands within the archipelago, and between these islands and the MIZ, they do not seem to swim south to reach their traditional denning islands in autumn. Offshore bears have the option of denning in Franz Josef Land, the Russian archipelago east and north of Svalbard, and an observed reduction in the number of dens in the eastern parts of Svalbard is thought to reflect an ongoing shift in preferred denning areas^34^. Offshore females with COYs that have denned in Svalbard may at some point have to choose between staying locally in Svalbard the first summer after denning, or risking losing their offspring on a long swim, as the pack ice continues to move farther away from Svalbard earlier in the summer. Local polar bears staying in Svalbard will face somewhat different challenges when dealing with shorter seasons with sea ice: reduced extent and duration of traditionally good seal hunting habitat, altered prey availability and access. Individuals that master aquatic stalking or other specialized, alternative hunting strategies may have an advantage in the coming decades.

When and how polar bears undertake swimming and diving is an integral part of their overall ecological strategy, which includes hunting strategies (or, more broadly, diet), energetics, reproductive strategies and risk management. Any extra energetic costs incurred via swimming, diving, or walking long distances, might be compensated for by enhanced access to better hunting areas or other types of food resources, higher hunting success, or higher quality denning or mating areas. This study quantified the amount of swimming done by female polar bears in the Barents Sea subpopulation, with the results indicating that it may be a more important behaviour than previously thought, especially for some individuals. This study did not attempt to discern or disentangle impacts on fitness of the various strategies used by the polar bears. Some strategies employed by Svalbard bears involve more swimming and diving, or increased likelihood of long distance swimming, than others. As the sea ice in the Arctic and in the Barents Sea in particular, continues to change and decline, the energetics costs, feasibility or risks associated with each strategy may be shifting. This study indicates that in the face of these changes, polar bears have the possibility to include extensive swimming in their strategy. It may become an important skill that will allow them to persist in an altered habitat - but only if the overall strategy is and remains profitable.

## Methods

This study was based on data from 57 adult female polar bears that were captured in Svalbard and equipped with various biologging devices with saltwater switches and pressure sensors that detect marine immersions and diving depths (Table 1; additional details in Appendix 1). These devices included two polar bear collar types with integrated sensors. Firstly, custom-built Sea Mammal Research Unit (SMRU) collars (n = 19; 2005–2006; SMRU, St Andrews, Scotland) that reported time spent in water and dive depth (using a pressure sensor) via Argos (System Argos, Toulouse, France) and secondly, Telonics collars (n = 31; 2015–2016; Telonics, Meza, AZ, USA) that reported GPS locations and time spent in the water directly via Iridium satellites. Additionally, Mk-9 Time-Depth Recorders (TDRs) from Wildlife Computers (N = 29; 2010–2017, Wildlife Computers, Redmond, WA, USA) were glued onto various satellite-linked collars (multiple makes, with collars providing GPS locations used in conjunction with the TDR data: N = 9). The TDRs are archival tags that need to be retrieved to access the data, a sample size of 29 resulted from 101 deployments. In all these various designs the saltwater switch was located low on the collar so that it was submerged when a polar bear was swimming normally.

Captures of polar bears for collar deployments and retrievals followed standard protocols (Stirling *et al*. 1989) and occurred mainly in April, but also in some years during March, May, August and September. Some individuals were instrumented in more than one year with different combinations of the equipment described above (consecutively or simultaneously). All animal handling protocols were approved by the Norwegian Animal Research Authority. The work was carried out in accordance with the relevant guidelines and regulations and under the permit of the Governor of Svalbard.

### General aquatic behaviour

Time spent in water and the probability of swimming were explored in relation to season, reproductive status (whether females are accompanied by cubs, and the age of the cubs), and offshore or local space use strategies. The descriptive summaries presented are based on different subsets of the data, provided by the different tag types described above. Hence, the sample sizes vary for the different analyses. Mean values of time spend in water were calculated monthly for each polar bear, but never based on fewer than five days of data. Three polar bears were not included in any of the analyses based on monthly means because their short or intermittent data records did not meet this criterion for any months. Dive depths are only shown from the six polar bears with SMRU collars that were recaptured and whose complete diving record could be downloaded. A recorded swim with a maximum depth indicating the polar bear had been fully submerged (e.g. 2 m) could be the result of a single dive or a bout of diving. Daily maximum depths were used in the summary of dive depths, as these gave a standardized measure against time.

Polar bear reproductive status, i.e. whether collared females had cubs and the age of the cubs, was determined at the time of capture and instrumentation, and that status was applied to the data from the time of capture until March of the following year. Females captured in April that had been in a maternity den that winter had COYs, which are about 4 months old during the spring tagging campaigns. Alternatively, females could be alone or accompanied by yearling cubs (about 16 months old). Females accompanied by two-year-olds (about 28 months old) were classified in this study as being alone, because mothers and cubs separate around this time. Some females with COYs and yearlings undoubtedly lost their cubs at some time during the year, but the timing of such a potential cub-loss was not known. Any instrumented females that were recaptured post-tag-deployment with yearling(s) or two-year-old(s), were known to have been accompanied by COY(s) or yearling(s) during the previous year. Time in water was compared across polar bears of different reproductive status by month, and statistical inference was based on a simple linear model with cub(s) age as the single predictor using data from each month. An initial comparison of polar bears with yearlings and polar bears with no cubs was made, to explore whether they could be pooled in a comparison against females with COYs. The comparison was limited to spring and summer months as sample size was small for the autumn months.

Monthly time in water was compared for the two main behavioural strategies that exist in the subpopulation^16,17^. Whether polar bears stay coastal or go offshore was classified by visual inspection of the tracking data. Fifteen polar bears were classified as ‘offshore’ based on having made at least one offshore excursion at some point during the period of tracking (available tracking records for individual polar bears prior to the current study period were also included in making this classification). Thirty-four polar bears were classified as ‘local’ because they did not make offshore excursions. Five polar bears could not be classified because their tracks did not span an entire summer.

Example tracks with location and time in water for three polar bears (N26241, N23479, and N23980) were chosen to illustrate individual strategies that involve swimming to different degrees. These three polar bears also showed three different types of swimming: coastal swimming at glacier fronts, likely associated with hunting, coastal swimming that was directional, and swimming during transits between land and the MIZ. Long-distance swims are described with distances and swimming durations. Because GPS locations were not acquired when a polar bear was in water (antenna submerged), locations during directional movements (transit trips and coastal trips) were interpolated between two known GPS positions, and these were considered the start and end of each trip.

### Modelling swimming in relation to sea ice concentration

How swimming behaviour varied with sea ice concentration when polar bears were not close to shore was modelled using generalized additive mixed effect models (GAMMs). The models were fitted to a subset of the data for which both GPS-tracks and time-in-water data were available. Time in water was considered within 2 h periods, to correspond with the time step of the GPS tracks. The 2 h period was centred on the time of the locations, and was either the mean of two hourly periods reported by the Telonics collars or the proportion of TDR measurements within a 2 h period that were wet. If both Telonics and TDR data for time in water were available for the same 2 h period, the Telonics data was used. Swimming data were allocated to geographical areas according to GPS positions from the collars. Tracks were linearly interpolated to fill gaps shorter than 48 h. This interval was chosen to avoid long periods of interpolation, while allowing for moderate gaps in the data record during long swims, as these often coincided with extended time spent in the water. None of the gaps longer than 48 h were associated with long swims, so this cut-off did not exclude any long swims from the analyses.

The data used in the analysis of sea ice concentration were screened according to the following criteria: sea ice concentration greater than 15%; minimum 6.25 km away from the bigger islands of Svalbard (Spitsbergen, Edgeøya, Barentsøya and Nordaustlandet); and minimum 1 km away from other islands within the archipelago. Sea ice concentration was extracted from daily 12.5 km resolution, 5-day median sea ice concentration data downloaded from ICDC - Product from University of Hamburg (http://icdc.zmaw.de/daten/cryosphere/seaiceconcentration-asi-ssmi.html). The dataset was limited to the period 1 March – 13 August, as there was little swimming data from other times of the year fulfilling the screening criteria. Distance from land was extracted using polygon files of the islands in Svalbard, from Norwegian Polar Institute archives (http://data.npolar.no). Altogether, 11,890 locations (1,946 of these interpolated) from 18 different polar bears were used in the offshore swimming models.

GAMMs were chosen to model possible nonlinearities in how swimming depended on season or sea ice concentration. Responses considered were proportion time in the water and whether a polar bear was wet more than some threshold duration (60 min, 10 min, 1 min, at all) in a 2 h period. These response variables were modelled with the same model structure, a spline for sea ice concentration as the main predictor variable, and a spline for Julian day to control for seasonality. The error structure included polar bear identity as a random effect on the intercept, and an autoregressive correlation with a lag of 1 (corAR1) to remove autocorrelation from the residual errors. Residual errors were assumed to follow a binomial distribution. The models were fitted using the ‘gamm4’ package in R 3.2.2^35^. Predictions regarding how swimming varied with sea ice concentration were made for the beginning, middle and end of the modelled time period (Julian days 61, 152, 225 (i.e. 1 March, 1 June and 13 August)).

### Data availability

The datasets analysed during the current study are available in the Norwegian Polar Data Centre repository [10.21334/npolar.2019.a27b8358].

## Electronic supplementary material

Supplementary Information
