# Supplementary material for: Aquatic behaviour of polar bears (Ursus maritimus) in an increasingly ice-free Arctic
Source: Sci Rep. 2018 Jun 26;8:9677. doi: 10.1038/s41598-018-27947-4 (PMC6018667; doi:10.1038/s41598-018-27947-4)
Supplement: Supplementary file 1 — Supplementary Information [file 41598_2018_27947_MOESM1_ESM.pdf]

Supplementary Information: Aquatic behaviour of polar bears (*Ursus maritimus*) in an increasingly ice-free Arctic

Karen Lone, Kit M. Kovacs, Christian Lydersen, Mike Fedak, Magnus Andersen, Philip Lovell, Jon Aars

APPENDIX 1: Detailed description of the devices deployed and data retrieval

A saltwater switch relies on saltwater's ability to conduct electricity to sense whether the tag is wet or dry. A tiny electrical current is established in a circuit that has a section where electricity must flow between two poles on the surface of the tag. When this is submerged in saltwater, the resistance is low because the saltwater forms part of the circuit, 'closing' the switch. When it is in air, the resistance is very high, because air has low conductivity. In all designs used in this study of polar bears, the saltwater switch was located low on the collar so that it was submerged when a polar bear was swimming normally.

In 2005-2006, nineteen SMRU (Sea Mammal Research Unit, St Andrews University, St Andrews Scotland) Argos-linked satellite-tracking collars were deployed on female polar bears in Svalbard. The collars used Satellite Relay Data Logger (SRDL) tag technology [1], designed to measure diving in marine mammals using an integrated saltwater switch to determine when the animal is in water and a pressure sensor that determines depth. Affiliated software was parametrized to identify a swimming event as starting when the sensor was immersed for 12 consecutive seconds and ending with the first dry second. For each period of swimming (a 'swim') multiple summary statistics were logged, including total duration and maximum depth. All information from individual swims, as well as 4 h summaries of dry time, wet time, and max depth was stored onboard the tag, and while deployed the tag attempted to transfer randomly selected batches of aquatic data via the Argos satellite system hourly. Each transmission also resulted in an attempt to estimate an Argos location, a calculation based on the Doppler Effect [2]. Six

SMRU collars were recovered upon recapture of the polar bears that carried them, allowing direct download of the complete records from these collars (678 ± 321 days, range 364-1278 days). The transmission of data from the SMRU collars through the Argos system was poor, so for the collars not recovered, the data records were often short (123 ± 114 days, range 0-359 days) and intermittent (data coverage 0.35 ± 0.33, range 0.007-0.98, records with more than 2 data points). The depth sensor integrated in the SMRU tags was designed for relatively shallow-diving marine mammals (0-20 m) so these tags provided reliable measures of maximum dive depth for each swimming event recorded (1.0 ± 1.3 m, range 0-13.9 m).

In 2015-2016, thirty-one Telonics (Telonics, Meza, AZ, USA) satellite-tracking collars were deployed on female polar bears in Svalbard. These devices had an integrated saltwater switch and recorded percent time spent in the water for every hour. These collars sent time in water, temperature, activity, and 12 GPS locations per day via the Iridium satellite system and thus polar bear recapture was not required to access the data. The algorithm underlying the aggregated hourly measure of time in water used quarter-second resolution. At least one measure of resistance lower than a threshold equivalent to ¼ the normal salinity of sea water in a second was required for it to be considered a wet second. A swimming event started when the collar recorded a minimum of five consecutive wet seconds, and ended with the first dry second. The Telonics collars provided time series with few data gaps. Durations of these records ranged from 40-736 days (320 ± 211 days).

During the period 2010-2016, Mk9 TDR archival tags from Wildlife Computers (Wildlife Computers, Redmond, WA, USA), were glued onto satellite-linked collars (multiple makes, 9 collars provided GPS locations that were used in conjunction with TDR data) deployed and retrieved on polar bears in Svalbard between 2010 and 2017. Following 101 deployments, thirty-six TDRs were physically recovered. Five instruments had corrupted memory files and on two the saltwater switch had failed, leaving 29 TDR

time series (372 ± 266 days, range 69-1239 days). These data records contained a saltwater switch reading every 10 s, as well as temperature, depth and light data at the same temporal resolution. The raw resistance values from the saltwater switch were rescaled between 1-256 before the data were stored. The data were aggregated to percentage time wet hourly, using a simple cut-off at 100 on the resistance scale to separate wet readings from dry readings. Exploratory analysis showed that the proportion time in water was not sensitive to the exact cut-off used, within the recommended range. Because the TDR tags were designed to be deployed on deep diving marine mammals, the pressure sensor was not sensitive enough to reliably pick out changes in depth of a few meters, so these records were used only for estimates of time spent in the water (and not diving).

Seven of the polar bears wearing Telonics collars with saltwater switches also had TDR records from the same time periods. There was a high correlation in recorded time spent in water between the two device types, with the correlation of hourly values ranging from 0.95 – 0.99 for the different sensors pairs. Monthly values of time spent in the water (range 0-0.24) were also highly correlated (0.99), with the Telonics collars recording slightly higher values than the TDRs (Appendix 1, Fig. A1). It was not possible to make similar comparisons with the SMRU collars, as their deployment did not overlap with either of the other sensor types.

Figure S1: Correlation between monthly time in water originating from simultaneous deployed Telonics collars and TDRs (based on six individuals, each contributing 3-12 months of data). The least squares regression line between proportion time in water from Telonics (y) and TDR (x) is y = 0.0070 + 0.99*x

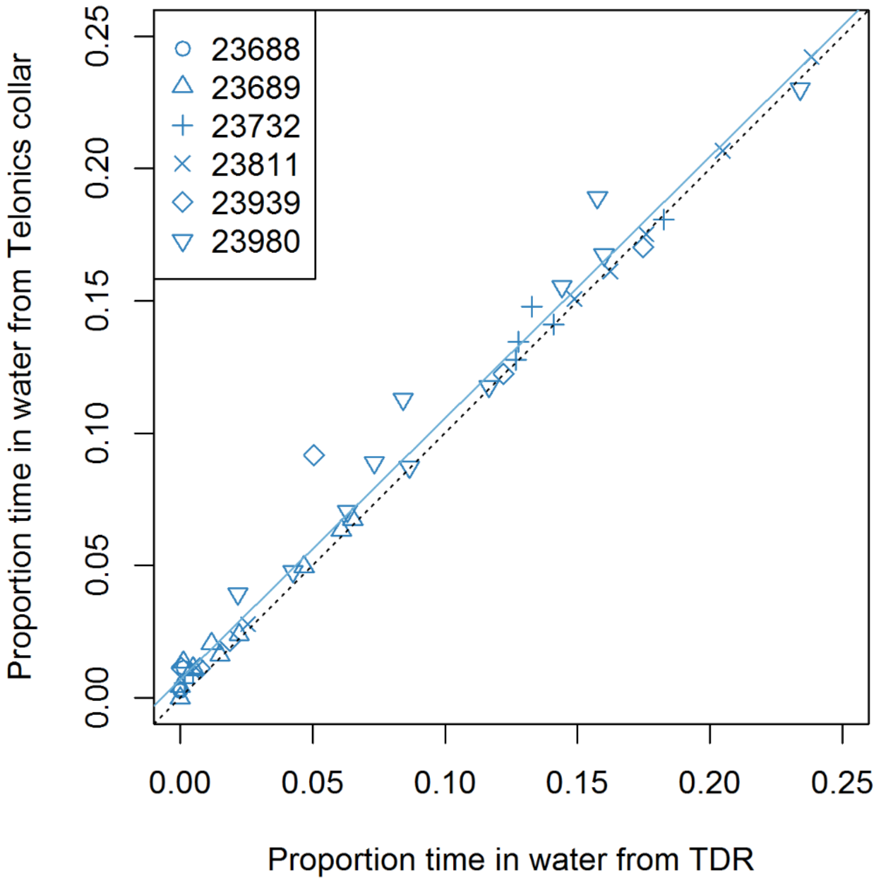

REFERENCES

1 Fedak, M., Lovell, P., McConnell, B. & Hunter, C. Overcoming the constraints of long range radio telemetry from animals: getting more useful data from smaller packages. *Integr. Comp. Biol.* **42**, 3-10 (2002).

2 Boehme, L. *et al.* Technical Note: Animal-borne CTD-Satellite Relay Data Loggers for real-time oceanographic data collection. *Ocean Sci.* **5**, 685-695 (2009).

APPENDIX 2: Additional detailed results

Table S1. Difference in mean monthly time spent in the water (proportion) by reproductive status of female polar bears or their space use strategy (simple linear model with reproductive status or space use strategy as the predictor variables)

	Estimated difference	SE	t-value	p-value
Yearling vs No Cubs				
Overall	0.01	0.01	0.5	0.65
Apr	-0.02	0.01	-1.6	0.12
May	-0.02	0.01	-2.2	0.04
Jun	-0.04	0.02	-1.9	0.06
Jul	0.01	0.03	0.3	0.80
Aug	0.07	0.02	2.8	0.01
Sep	0.04	0.04	1.1	0.28
COY vs no COY				
Overall	0.03	0.01	4.4	<0.001
Apr	0.02	0.01	3.5	<0.001
May	0.03	0.01	5.7	<0.001
Jun	0.04	0.01	3.8	<0.001
Jul	0.05	0.02	2.8	0.007
Aug	0.01	0.02	0.7	0.50

Sep	0.02	0.02	0.8	0.43
Offshore vs Local				
Overall	-0.01	0.01	-1.3	0.19
Jan	-0.01	0.02	-0.9	0.35
Feb	0.00	0.02	0.0	0.97
Mar	0.01	0.01	0.9	0.40
Apr	0.01	0.01	0.7	0.50
May	0.01	0.01	2.1	0.04
Jun	0.01	0.01	0.6	0.57
Jul	-0.04	0.02	-1.7	0.10
Aug	-0.02	0.02	-0.8	0.43
Sep	-0.02	0.03	-0.8	0.41
Oct	-0.02	0.02	-0.8	0.42
Nov	-0.01	0.02	-0.5	0.63
Dec	0.00	0.02	0.0	0.97
